# Enhancing the production of chlorophyll *f* in the cyanobacterium *Synechocystis* sp. PCC 6803

**DOI:** 10.1111/ppl.70169

**Published:** 2025-03-26

**Authors:** Man Qi, Henry N. Taunt, Martina Bečková, Zhi Xia, Joko P. Trinugroho, Josef Komenda, Peter J. Nixon

**Affiliations:** ^1^ Sir Ernst Chain Building‐Wolfson Laboratories, Department of Life Sciences Imperial College London London UK; ^2^ Institute of Microbiology of the Czech Academy of Sciences, Center Algatech Třeboň Czech Republic; ^3^ Present address: Baseimmune, BioScience Innovation Centre London UK; ^4^ Present address: Research Center for Genetic Engineering, Research Organization for Life Science and Environment, National Research and Innovation Agency Cibinong Bogor Indonesia

## Abstract

One potential approach to improve the productivity of cyanobacteria and microalgae is to enhance photosynthetic efficiency by introducing far‐red absorbing pigment molecules (such as chlorophylls *f* and *d*) into the photosynthetic apparatus to expand the range of photosynthetically active radiation. We have shown previously that expressing the ChlF subunit of *Chroococcidiopsis thermalis* PCC 7203 in the model cyanobacterium *Synechocystis* sp. PCC 6803 (*Syn*6803) is sufficient to drive the production of chlorophyll *f* (Chl *f*), but only to low levels (0.24% Chl *f*/Chl *a*). By using the strong P_*cpc560*_ promoter and an N‐terminal truncated derivative of ChlF, we have been able to increase the yield of Chl *f* in white light by over 30‐fold to about 8.2% Chl *f*/Chl *a*, close to the level displayed by far‐red photoacclimated *C. thermalis* 7203. Additionally, we demonstrate that ChlF from *Fisherella thermalis* PCC 7521, like ChlF from *C. thermalis* 7203, assembles into a variant of the monomeric photosystem II (PSII) core complex termed the super‐rogue PSII complex when expressed in *Syn*6803. This contrasts with the originally reported formation of a ChlF homodimeric complex in *Synechococcus* sp. PCC 7002. Overall, our work is an important starting point for mechanistic and structural studies of super‐rogue PSII and for incorporating Chl *f* into the photosynthetic apparatus of *Syn*6803.

## 1 INTRODUCTION

Photoautotrophs, such as algae and cyanobacteria, have attracted significant interest as low‐cost, carbon‐neutral systems for the production of high‐value and commodity chemicals (Bühler and Lindberg, 2023; Fabris et al., 2020; Li et al., 2023; Naduthodi et al., 2021). However, the efficiency of these systems is currently constrained by the natural limitations of solar‐to‐chemical energy conversion in oxygenic photosynthesis (Kruse et al., 2005).

One promising avenue to enhance the light‐usage efficiency of photosynthetic organisms is to expand the range of photosynthetically active radiation to include the far‐red region of the solar spectrum (700–800 nm), which potentially increases photon capture for photosynthesis by up to 19% (Blankenship and Chen, 2013; Chen and Blankenship, 2011). A breakthrough in this area has been the discovery of far‐red light (FRL)‐absorbing pigments, chlorophyll *f* (Chl *f*) and chlorophyll *d* (Chl *d*), in specific cyanobacterial species (Chen et al., 2010; Gan et al., 2014a; Miyashita et al., 1996). Potentially, incorporating these pigments into the photosynthetic apparatus could significantly boost the capacity of oxygenic photosynthesis in FRL‐rich environments, such as the interior of microalgal cultures or the lower regions of the plant canopy (Cardona et al., 2018; Ort et al., 2015).

Chl *f* synthesis requires the ChlF protein (Ho et al., 2016), also referred to as PsbA4 or super‐rogue D1 (srD1), a variant of the D1 subunit of photosystem II (PSII) (Murray, 2012). Unlike D1, ChlF lacks crucial residues for binding the Mn_4_CaO_5_ cluster required for light‐driven water oxidation, though it retains residues essential for light‐induced charge separation (Cardona et al., 2015; Murray, 2012). While heterologous expression of ChlF drives Chl *f* production in cyanobacteria unable to produce Chl *f* naturally, the yields obtained in initial experiments were over 40‐fold less than the native systems (Ho et al., 2016; Trinugroho et al., 2020) and a potential bottleneck for re‐engineering the photosynthetic apparatus. Additionally, debate continues over whether ChlF functions as a homodimer (Ho et al., 2016; Shen et al., 2019) or is part of a monomeric PSII‐like srPSII complex in which srD1 replaces D1 (Trinugroho et al., 2020). Here, we describe the enhanced production of Chl *f* in the model cyanobacterium *Syn*6803 using the strong P_*cpc560*_ promoter (Zhou et al., 2014) and a variant of ChlF encoded by *C. thermalis* 7203 with a shortened N‐terminus. We also confirm that strains producing high levels of Chl *f* still assemble ChlF into the srPSII complex, with no evidence of the formation of a ChlF homodimer.

## 2 MATERIALS AND METHODS

### 2.1 Strains and culture conditions

Two *Syn*6803 strains were used in this study: the glucose‐tolerant GT‐P wild‐type strain, denoted WT (Tichý et al., 2016), and a *psbA* triple‐deletion strain expressing His‐tagged CP47, denoted ΔPsbA (Debus et al., 2001). *Syn*6803 strains and their transformants were cultivated photoautotrophically on BG11 agar plates or mixotrophically by adding 5 mM glucose. Liquid BG11 cultures containing 5 mM glucose were grown in sterile, vented tissue culture flasks on an orbital shaker, as described in (Trinugroho et al., 2020). Antibiotics, zeocin (10–50 μg mL^−1^) and kanamycin (10–25 μg mL^−1^), were added as needed to both agar plates and liquid cultures. Cultures were grown in a temperature‐controlled room set at 29–30°C and illuminated by white light fluorescent light from above (Sylvania Grolux fluorescent tube T8 58W). Four different light intensities were used in this study: very low (<5 μmol photons m^−2^ s^−1^), low (2–10 μmol photons m^−2^ s^−1^), medium (20–35 μmol photons m^−2^ s^−1^) and medium‐high (35–50 μmol photons m^−2^ s^−1^), measured at the flask surface.

### 2.2 Construction of vectors and ChlF mutants

#### 2.2.1 Construction of pGT270_Flag‐ChlF^7521^ vectors and mutants

The pGT270_Flag‐ChlF^7521^ vector containing either a synthetic composite promoter and ribosome‐binding site from plasmid pGT321 (hereafter designated P_*gt321*_) (Taylor et al., 2021) or a P_*cpc560*_ promoter (Zhou et al., 2014) was assembled following the STEP Golden Gate assembly architecture (Taunt, in preparation). The vector features either the P_*gt321*_ or P_*cpc560*_ promotor for expression of an N‐terminal 3xFlag‐tagged ChlF from *Fisherella thermalis* PCC 7521 (Flag‐ChlF^7521^), along with a T_*L3S2P21*_ terminator containing translation enhancing properties (Figure S1). The expression cassette is coupled with the *Streptoalloteichus hindustanus* bleomycin‐resistance gene marker (*Ble* ^*R*^) in addition to a kanamycin‐resistance marker (*Kan* ^*R*^), and all three genes are flanked by homology arms targeting the *ssl0410* locus (Taylor et al., 2021). The pGT270 backbone, P_*gt321*_ promoter (sequence shown in Table S1) and T_*L3S2P21*_ terminator were a generous gift from Dr. John Heap (University of Nottingham), and the *Ble* ^*R*^ cassette was a generous gift from Dr. Lydia Mapstone via Prof Saul Purton (University College London). The P_*cpc560*_ promotor and the *chlF* ^*7521*^ gene were synthesized *de novo* by Twist Bioscience (sequences shown in Table S1). The pGT270_Flag‐ChlF^7521^ vectors were used to transform the *Syn*6803 WT or ΔPsbA strain, generating the P_*gt321*__Flag‐ChlF^7521^/WT*, P_*cpc560*__Flag‐ChlF^7521^/WT* and P_*cpc560*__Flag‐ChlF^7521^/ ΔPsbA* mutants (Table 1).

**TABLE 1:** List of ChlF mutants investigated in this study.

Mutants	Vector transformed	Promoter	Gene of interest	Integration locus	Resistance marker
P_*gt321*__Flag‐ChlF^7521^/WT*	pGT270_P_*gt321*__Flag‐ChlF^7521^	P_*gt321*_	*Flag‐chlF* ^*7521*^	*ssl0410*	*Ble* ^*R*^ & *Kan* ^*R*^
P_*cpc560*__Flag‐ChlF^7521^/WT*	pGT270_P_*cpc560*__Flag‐ChlF^7521^	P_*cpc560*_	*Flag‐chlF* ^*7521*^	*ssl0410*	*Ble* ^*R*^ & *Kan* ^*R*^
P_*cpc560*__Flag‐ChlF^7521^/ΔPsbA*	pGT270_P_*cpc560*__Flag‐ChlF^7521^	P_*cpc560*_	*Flag‐chlF* ^*7521*^	*ssl0410*	*Ble* ^*R*^ & *Kan* ^*R*^
P_*psbA2*__ChlF^7203^/ΔPsbA	pPD_ChlF^7203^	P_*psbA2*_	*chlF* ^*7203*^ *_co*	*psbA2*	*Kan* ^*R*^
P_*psbA2*__NtChlF^7203^/ΔPsbA	pPD_NtChlF^7203^	P_*psbA2*_	*NtchlF* ^*7203*^ *_co*	*psbA2*	*Kan* ^*R*^
P_*cpc560*__ChlF^7521^/ΔPsbA	pFly_ChlF^7521^	P_*cpc560*_	*chlF* ^*7521*^	*ssl0410*	*Ble* ^*R*^
P_*cpc560*__Flag‐ChlF^7521^/ΔPsbA	pFly_Flag‐ChlF^7521^	P_*cpc560*_	*Flag‐chlF* ^*7521*^	*ssl0410*	*Ble* ^*R*^
P_*cpc560*__NtChlF^7203^/ΔPsbA	pFly_NtChlF^7203^	P_*cpc560*_	*NtchlF* ^*7203*^	*ssl0410*	*Ble* ^*R*^
P_*cpc560*__Flag‐NtChlF^7203^/ΔPsbA	pFly_Flag‐NtChlF^7203^	P_*cpc560*_	*Flag‐NtchlF* ^*7203*^	*ssl0410*	*Ble* ^*R*^
P_*cpc560*__ChlF^7521^/WT	pFly_ChlF^7521^	P_*cpc560*_	*chlF* ^*7521*^	*ssl0410*	*Ble* ^*R*^
P_*cpc560*__NtChlF^7203^/WT	pFly_NtChlF^7203^	P_*cpc560*_	*NtchlF* ^*7203*^	*ssl0410*	*Ble* ^*R*^

* is to distinguish the mutants transformed with pGT270_Flag‐ChlF^7521^ vectors (*Ble* ^*R*^ & *Kan* ^*R*^) from that transformed with pFly‐ChlF vectors (*Ble* ^*R*^); co, codon‐optimized; *Ble* ^*R*^, bleomycin‐resistance marker; *Kan* ^*R*^, kanamycin‐resistance marker.

#### 2.2.2 Construction of pPD_(Nt)ChlF^7203^ vectors and mutants

The pPD_(Nt)ChlF^7203^ vectors were constructed to express either a full‐length ChlF from *C. thermalis* 7203 (ChlF^7203^) or a N‐terminal truncated ChlF^7203^ derivative lacking residues V2 to V23 (NtChlF^7203^) using the native P_*psbA2*_ promoter in *Syn*6803. The *chlF* ^*7203*^ gene, codon‐optimized and synthesized *de novo* by Twist Bioscience to facilitate expression in chloroplast of *Chlamydomonas reinhardtii* but with suitability extended to *Syn*6803 (sequence shown in Table S1), was amplified in both full length (*chlF* ^*7203*^_co) and truncated (*NtchlF* ^*7203*^_co) versions using distinct sets of primers (Table S2). Both sequences were then assembled into a NdeI and BglII digested pPD Flag vector (Trinugroho et al., 2020) via in‐fusion cloning (Zhu et al., 2007). The resulting vectors were used to transform the *Syn*6803 ΔPsbA strain, generating P_*psbA2*__ChlF^7203^/ΔPsbA and P_*psbA2*__NtChlF^7203^/ΔPsbA (Table 1).

#### 2.2.3 Construction of pFly vectors and mutants

The pFly vector was designed as a single‐step Golden Gate drop‐in vector to allow for direct cloning of a coding sequence (CDS) into a transformation‐ready high‐expression plasmid. It was constructed from the parental vector pGT270_P_*cpc560*__Flag‐ChlF^7521^ by the removal of the original CDS and a number of unnecessary elements, such as the additional kanamycin‐resistance marker (*Kan* ^*R*^) and the f1 phage origin. SapI‐type IIS restriction sites were added for the single‐step cloning of CDS directly into the vector, and the *tsPurple* chromoprotein marker was added inside the drop‐in restriction sites for screening of colonies following Golden‐Gate cloning.

The *chlF* ^*7521*^ gene was synthesized *de novo*, and the *NtchlF* ^*7203*^ gene (sequence shown in Table S1) was amplified from *C. thermalis* 7203 genomic DNA to include SapI restriction sites with the 5′ ATG and 3′ TAA fusion sites, allowing scar‐free integration of CDSs into the pFly vector. When the inclusion of an N‐terminal tag was required, the CDS part was modified to include a 5′ AGT fusion site instead of an ATG, allowing the insertion of an ATG–AGT N‐terminal tag part. The resulting pFly vectors with ChlF variants, including ChlF^7521^, NtChlF^7203^, Flag‐ChlF^7521^ and Flag‐NtChlF^7203^ were used to transform the *Syn*6803 ΔPsbA or WT strains, generating P_*cpc560*__ChlF^7521^/ΔPsbA, P_*cpc560*__Flag‐ChlF^7521^/ΔPsbA, P_*cpc560*__NtChlF^7203^/ΔPsbA, P_*cpc560*__Flag‐NtChlF^7203^/ΔPsbA, P_*cpc560*__ChlF^7521^/WT and P_*cpc560*__NtChlF^7203^/WT (Table 1).

### 2.3 Room‐temperature spectroscopy

Room‐temperature fluorescence of whole cells and proteins (concentrations corresponding to 3–5 μg mL^−1^ Chl *a*) were recorded using a Fluoromax plus spectrofluorometer (Horiba Scientific). The excitation wavelength was set to 440 nm (2 nm slit width) to preferentially excite chlorophylls and fluorescence spectra were recorded in the 600–800 nm range (2 nm slit width). Room‐temperature absorption spectra and optical density at 750 nm (OD_750 nm_) of cells were recorded using a UV‐3000 spectrophotometer (Shimadzu).

### 2.4 In‐gel 77 K fluorescence

In‐gel fluorescence was recorded using a custom device constructed by Dr. Geoffry Davis, based on the method outlined by Lucker et al. (2017). Excitation was achieved with a 450 nm LED (Luxeon Rebel Royal Blue, Luxeon Star LEDs). The excitation light was directed to the sample through a bifurcated fibre optic, with the merged end of the fibre optic positioned perpendicularly to the gel. This setup allowed simultaneous delivery and collection of excitation and fluorescence emission. Fluorescence emission was filtered using an FGL 495 filter to eliminate excitation wavelengths. For low‐temperature measurements at 77 K, the gel was cooled in a liquid nitrogen bath. An XY translation stage facilitated the precise positioning of the gel under the merged end of the fibre optic.

### 2.5 Flag‐affinity purification

For isolation of Flag‐ChlF^7521^ complexes, 2 x 10 L P_*cpc560*__Flag‐ChlF^7521^/ΔPsbA cultures were cultivated in 2 x 20 L carboys under medium‐high white light irradiance (35–50 μmol photons m^−2^ s^−1^) until reaching an OD_750_ of approximately 1.6. After pelleting, cells were washed and resuspended in buffer A (50 mM MES/NaOH, pH 6.5, 1.2 M glycine betaine, 5 mM CaCl_2_, 10 mM MgCl_2_, 10% (v/v) glycerol). Cell disruption was performed using a CF1 cell disrupter (Constant Systems), followed by centrifugation (Avanti J‐15R, 5000 *g*, 5 min) to remove unbroken cells and cellular debris. Thylakoid membranes were then pelleted by centrifugation (Beckman Optima XPN‐100; Type 45 Ti, 167,900 *g*, 25 min) and resuspended in buffer B (50 mM MES/NaOH, pH 6.5, 1.2 M glycine betaine, 20 mM CaCl_2_, 10 mM MgCl_2_, 0.5 M mannitol) to a Chl *a* concentration of 0.5–1 mg mL^−1^. Membranes were solubilized for 30 min with 1% (w/v) n‐dodecyl‐β‐D‐maltoside (DDM) at 4°C on a rotating wheel in the dark. Following centrifugation for 25 min at 167,900 *g* (Beckman Optima XPN‐100; Type 45 Ti) to remove insolubilized membrane particles, the supernatant was incubated with Pierce™ Anti‐Flag Affinity Resin (ThermoFisher Scientific) for 1 h at 4°C in the dark. Proteins bound to the anti‐Flag resin were washed three times with 2 resin volumes of buffer B containing 0.04% (w/v) DDM and then eluted from the resin using buffer B containing 0.04% (w/v) DDM and 150 μg mL^−1^ 3xFlag peptide (Sigma‐Aldrich).

### 2.6 Protein electrophoresis and immunoblotting

Two‐dimensional (2D) gel electrophoresis and immunoblotting were performed as described by Trinugroho et al. (2020). Briefly, solubilized membranes or isolated proteins were loaded on a 4–14% (w/v) polyacrylamide clear‐native (CN) PAGE gel to facilitate the separation of complexes. Following this, the gel was scanned to capture the room‐temperature fluorescence image. Subsequently, the complexes were denatured and loaded onto a 12–20% (w/v) polyacrylamide SDS‐PAGE gel (with 7 M urea) for the separation of individual subunits. For the one‐dimensional SDS‐PAGE gels shown in Figures S1 and S2, denatured proteins were loaded onto a Novex 14% Tris‐Glycine gel (Invitrogen). Proteins were visualized through staining with Coomassie Blue or SYPRO Orange and detected by immunoblotting. Primary antibodies utilized in this study included anti‐FLAG (Abgent, cat. no. AP1013A), anti‐D2 and anti‐CP47 (Trinugroho et al., 2020), anti‐Ycf48 (Zhao et al., 2023), anti‐CyanoQ (Michoux et al., 2014) and Psb28‐1 (Dobáková et al., 2008).

### 2.7 Pigment quantification

Pigments were extracted from cells or isolated proteins with 100% methanol. Chl *a* content was determined spectrophotometrically using a UV‐3000 spectrophotometer (Shimadzu) according to the equations outlined by Ritchie (2006). Chl *f* quantification involved injecting pigment extracts into an HPLC machine (Agilent‐1200). Pigments were separated by a reversed‐phase column (Poroshell EC‐C18) using the buffer systems described in method MY2 in Ho et al. (2016). The HPLC program started with 100% solvent A (methanol:ethyl acetate:water = 88:10:2) for 10 min at a flow rate of 0.8 mL min^−1^. Subsequently, the ratio of solvent B (methanol:ethyl acetate:water = 48:50:2) linearly increased to 20% in 5 min, then increased to 100% in 10 s and then held at 100% for 5 min. A post‐gradient elution of 5 min was added for the recovery of the initial solvent system. Spectra were recorded in the range of 400–800 nm, and chromatograms were obtained at 665 nm and 707 nm to quantify Chl *a* and Chl *f*, respectively. The Chl *f* to Chl *a* molar ratio was estimated by integrating their Qy absorption peaks (Chl *f* at 707 nm and Chl *a* at 665 nm) (Li et al., 2012) and expressed as a percentage.

## 3 RESULTS

### 3.1 Using the P_*cpc560*_ promoter to express ChlF in *Syn*6803

The heterologous production of Chl *f* in cyanobacteria has so far been demonstrated for two widely used model cyanobacteria: *Synechococcus* sp. PCC 7002 (hereafter *Syn*7002) and *Syn*6803. Integration of the *chlF* gene from *Chlorogloeopsis fritschii* PCC 9212 (encoding ChlF^9212^) into an endogenous plasmid in *Syn*7002 (with expression driven by the *Synechocystis* P_*cpcBA*_ promoter) led to the accumulation of 0.059% Chl *f*/Chl *a* (Ho et al., 2016) and insertion of the *chlF* gene from *Chroococcidiopsis thermalis* PCC 7203 (encoding ChlF^7203^) into the *psbA2* locus of a D1 deletion strain of *Syn*6803 (driven by the native P_*psbA2*_ promoter) led to 0.24% Chl *f*/Chl *a* (Trinugroho et al., 2020). More recently, Shen et al. (2019) reported the increased production of Chl *f* in WT *Syn*7002 by expressing ChlF from *Fisherella thermalis* PCC 7521 (ChlF^7521^) (<0.5% Chl *f*/Chl *a* in low‐intensity white light) and improved levels of 3–4% Chl *f*/Chl *a* using a *Syn*7002 mutant lacking the PSII D2 subunit and grown in far‐red‐enriched light.

To check whether ChlF^7521^ can also function in *Syn*6803, we heterologously expressed a 3xFlag‐tagged derivative of ChlF^7521^ in *Syn*6803 and explored two different promoters for ChlF^7521^ expression: the low‐burden synthetic P_*gt321*_ promoter (Taylor et al., 2021) and the strong P_*cpc560*_ promoter (Zhou et al., 2014). Our results confirmed that the P_*cpc560*_ promoter conferred substantial advantages for both ChlF^7521^ expression and Chl *f* production in WT *Syn*6803 (Figure S1). In line with the observations reported by Shen et al. (2019) for *Syn*7002, a greater than two‐fold increase in Chl *f* levels was achieved by cultivating the mutants under low (2–10 μmol photons m^−2^ s^−1^) rather than medium (20–35 μmol photons m^−2^ s^−1^) irradiances of white light, with a value of 2.0–2.2% Chl *f*/Chl *a* obtained when expressed in either the WT or in the ΔPsbA strain lacking D1 (Figure S2).

### 3.2 High‐level Chl *f* production using an N‐terminally truncated ChlF from *C. thermalis* 7203

ChlF and D1 exhibit notable differences in primary structure in the N‐terminal regions of the proteins (Figure S3), with, for instance, ChlF^7203^ predicted to have an N‐terminal extension of 22 amino‐acid residues compared with D1 (Trinugroho et al., 2020). Expressing an N‐terminally truncated derivative of ChlF^7203^ (NtChlF^7203^) lacking 22 residues at the N‐terminus (ΔV2‐V23) in the ΔPsbA mutant using the native P_*psbA2*_ promoter (P_*psbA2*__NtChlF^7203^/ΔPsbA) led to a dramatic increase in Chl *f* levels compared to a mutant expressing full length ChlF^7203^ (P_*psbA2*__ChlF^7203^/ΔPsbA) (1.4 vs. 0.1% Chl *f*/Chl *a*) (Figure S4). Note the *chlF* ^*7203*^ and *NtChlF* ^*7203*^ genes used here were codon‐optimized, yet demonstrated comparable Chl *f* production capacities to that of non‐codon‐optimized versions examined by Trinugroho (2020).

### 3.3 Construction of the pFly vector for driving ChlF expression

**FIGURE 1:**
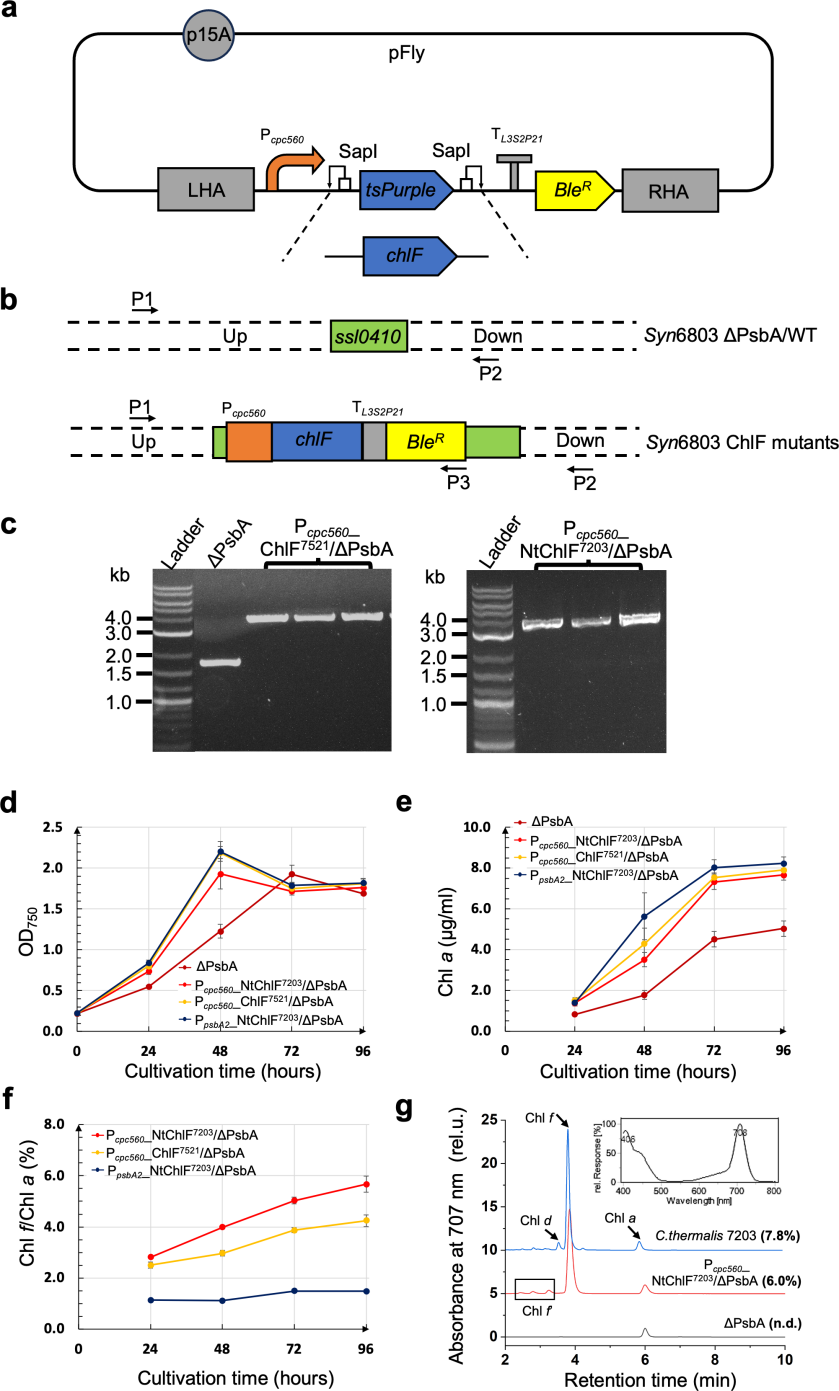
**Testing Chl *f* production in various ChlF mutants. a**, Cartoon illustrating the composition of the pFly vector and introduction of *chlF* genes into the pFly vector. LHA, left homologous arm; RHA, right homologous arm; *Ble* ^*R*^, bleomycin‐resistance gene; *tsPurple*, *tsPurple* chromophore cassette. **b**, The *ssl0410* locus of the *Syn*6803 ΔPsbA strain and resulting ChlF mutants after transformation of the pFly‐ChlF vectors into *Syn*6803. Binding sites of the primers (Table S2) used for PCR genotyping are indicated. **c**, Agarose gel of PCR fragments confirming the genotypes of ChlF mutants. The predicted sizes of the PCR fragments from the ChlF mutants are 3.5–3.6 kb, while that from ΔPsbA is 1.7 kb. **d**,**e**,**f**, Changes in cell density (OD_750_) (**d**), Chl *a* content in cells (**e**), and Chl *f*/Chl *a* levels in cells (**f**) of the various ChlF mutants grown in triplicate during continuous cultivation under low light (5–10 μmol photons m^−2^ s^−1^). The differences in Chl *f* content in all three strains at 96 h are statistically significant as determined using one‐tailed t‐tests (p‐value <0.05) **g**, HPLC elution profiles at 707 nm for pigments extracted from ΔPsbA, one of the cultures of the P_*cpc560*__NtChlF^7203^/ΔPsbA mutant at 72 h, and *C. thermalis* 7203. The calculated Chl *f*/Chl *a* levels in different cells are indicated in bold parentheses. The black arrows indicated the positions of Chl *d*, Chl *f*, and Chl *a* peaks in *C. thermalis* 7203. Inset is the spectrum of the Chl *f* peak in the P_*cpc560*__NtChlF^7203^/ΔPsbA mutant. The three small peaks in the black frame, labeled as Chl *f’*, had a similar in‐line absorption spectrum as Chl *f* and have been suggested to be Chl *f* species esterified with geranylgeraniol and its dihydro and tetrahydro derivatives rather than phytol (Shen et al., 2019). rel.u., relative unit; n.d., not detected.

To investigate further the Chl *f* production capabilities of various ChlF variants in *Syn*6803, we designed a one‐step drop‐in vector named pFly, featuring the high‐expression promoter P_*cpc560*_ and the translation‐enhancing terminator T_*L3S2P21*_. pFly includes flanking regions for insertion into the *ssl0410* locus of *Syn*6803 genomic DNA, the *Streptoalloteichus hindustanus* bleomycin‐resistance gene (*Ble* ^*R*^) for zeocin selection (Hu et al., 2012), the *tsPurple* chromophore cassette for “pink/white” screening of transformant colonies (Lazarus et al., 2019), and the low‐copy‐number origin p15a to mitigate *E. coli* toxicity issues (Figure 1a). SapI restriction sites flanking the *tsPurple* cassette facilitate the one‐step drop‐in of various *chlF* coding sequences using SapI Golden‐Gate cloning (Taylor et al., 2019).

To explore whether expressing NtChlF^7203^ using the P_*cpc560*_ promoter would further enhance Chl *f* production and to directly compare the Chl *f* production capabilities of NtChlF^7203^ and ChlF^7521^, the corresponding *chlF* genes were cloned into the pFly vector and used to transform the ΔPsbA strain of *Syn*6803 to generate two mutants: one expressing NtChlF^7203^ (P_*cpc560*__ NtChlF^7203^/ ΔPsbA) and the other ChlF^7521^ (P_*cpc560*__ ChlF^7521^/ ΔPsbA) (Figure 1b, c).

Both mutants, together with the P_*psbA2*__NtChlF^7203^/ΔPsbA mutant, were grown in BG11 medium with 5 mM glucose under continuous low light (5–10 μmol photons m^−2^ s^−1^) and the accumulation of Chl *a* and Chl *f* was monitored in cells using absorbance methods and reversed‐phase HPLC.

The Chl *a* content in all mutants increased rapidly during the first 2–3 days of cultivation, slowing down as cells entered the stationary phase (Figure 1d, e). In contrast, Chl *f* levels in cells increased consistently throughout cultivation (Figure 1f). The P_*cpc560*__NtChlF^7203^/ΔPsbA mutant exhibited the highest Chl *f* productivity at the end of cultivation (5.7 ± 0.3% Chl *f*/Chl *a*), followed closely by the P_*cpc560*__ChlF^7521^/ΔPsbA mutant (4.2 ± 0.2% Chl *f*/Chl *a*), while the P_*psbA2*__NtChlF^7203^ mutant showed a lower accumulation of (1.5 ± 0.1% Chl *f*/Chl *a*). The production of Chl *f* was confirmed by its identical elution time and absorption spectrum to that of Chl *f* extracted from FRL‐adapted *C. thermalis* 7203 cells (Figures 1g; S5).

In other experiments conducted at lower light irradiances (<5 μmol photons m^−2^ s^−1^), Chl *f* levels reached 8.2% Chl *f*/Chl *a* in the P_*cpc560*__NtChlF^7203^/ΔPsbA mutant and 5.4% in the P_*cpc560*__ ChlF^7521^/ ΔPsbA strain when grown to stationary phase (OD_750_ at approximately 2) (Figure S5) ‐ a level comparable to that observed in FRL‐adapted *C. thermalis* 7203 cells and other FRL‐photoacclimated species (Gan et al., 2014a). For strains constructed in the WT background, mixotrophic and photoautotrophic growth was similar to WT in growth spot tests and levels of Chl *f* reached 3.7–4.2% Chl *f*/Chl *a* for the P_*cpc560*__ChlF^7521^/WT mutant and 4.8–7.4% Chl *f*/Chl *a* for the P_*cpc560*__NtChlF^7203^/WT mutant (Figure S6).

Overall, these results emphasize the benefits of using the P_*cpc560*_ promoter and the N‐terminal truncated variant of NtChlF^7203^ to produce Chl *f* in *Syn*6803. The reasons behind the improved performance of the NtChlF^7203^ variant remain to be determined but could be related to the levels of srPSII that accumulate.

### 3.4 Room‐temperature fluorescence as a rapid method to determine Chl *f* levels

Compared to white light (WL)‐grown cells containing only Chl *a*, FRL‐adapted *C. thermalis* 7203 cells exhibit additional absorption and fluorescence bands above 700 nm at room temperature which are attributed to the presence of FRL‐absorbing pigments, Chl *d* and Chl *f*, in the FRL‐photosystems, along with the synthesis of FRL‐allophycocyanins (FRL‐APCs) (Mascoli et al., 2022). In this study, we conducted room‐temperature fluorescence spectroscopy on various ChlF mutants and compared the results with their parental *Syn*6803 strains to assess whether the incorporation of Chl *f* in the mutants results in enhanced fluorescence above 700 nm.

Besides the ChlF mutants discussed above, two *Syn*6803 ΔPsbA mutants expressing N‐terminal 3xFlag‐tagged derivatives of NtChlF^7203^ (P_*cpc560*__Flag‐NtChlF^7203^/ΔPsbA) and ChlF^7521^ (P_*cpc560*__Flag‐ChlF^7521^/ΔPsbA) were generated (Figure S7), to allow the isolation of ChlF complexes.

**FIGURE 2:**
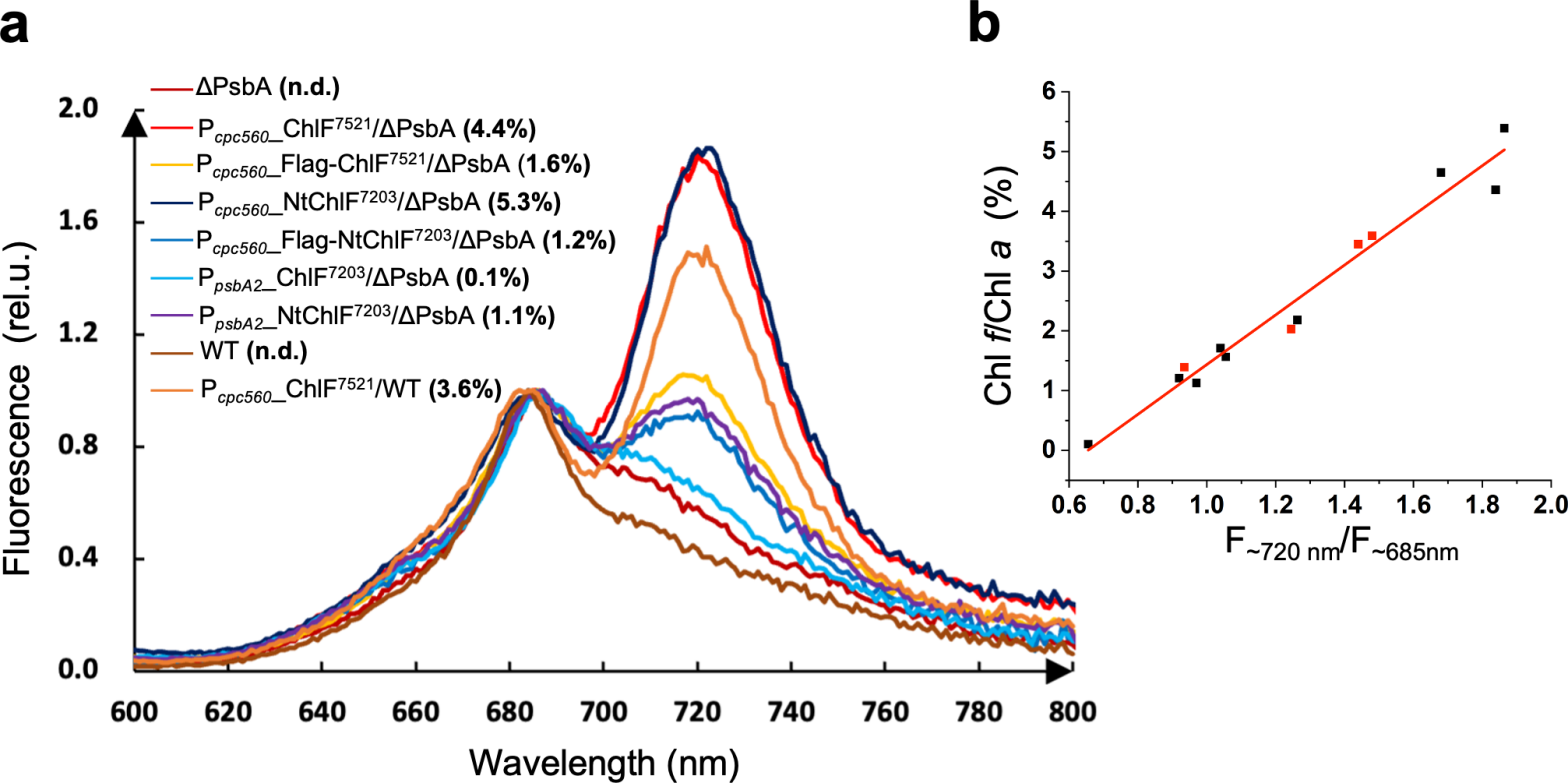
**Room‐temperature fluorescence analysis of cells. a**, Room‐temperature fluorescence spectra of various ChlF mutants. The excitation wavelength was set to 440 nm and spectra were normalized to the peak fluorescence at 680–685 nm. Lines with different colors represent spectra obtained from different ChlF mutants with respective Chl *f*/Chl *a* levels indicated in bold parentheses. n.d., not detected; rel. u., relative unit. **b**, Relation of the relative height of the ~720 nm fluorescence peak (F_~720 nm_/F_~685nm_) with the Chl *f*/Chl *a* levels detected in cells. Black dots were obtained from ΔPsbA mutants while the red dots were obtained from WT mutants.

All strains were grown in BG11 with 5 mM glucose under continuous low light (2–10 μmol photons m^−2^ s^−1^) and underwent room‐temperature fluorescence spectroscopy when the OD_750_ was around 2–3. Concurrently, the Chl *f* content in these mutants was quantified by reversed phase HPLC for comparative analysis. As shown in Figure 2a, most of the ChlF mutants showed an additional emission band centred at 717 ~ 723 nm compared with the parental ΔPsbA and WT strains, which is attributed to the presence of Chl *f*.

The P_*cpc560*__NtChlF^7203^/ΔPsbA and P_*cpc560*__ChlF^7521^/ΔPsbA mutants with higher Chl *f* content (5.3% and 4.4% Chl *f*/Chl *a*, respectively) exhibited a more pronounced amplitude at ~720 nm compared to their Flag‐tagged derivatives (1.2 and 1.6%, respectively), P_*cpc560*__ChlF^7521^/WT (3.6%) and P_*psbA2*__NtChlF^7203^/ΔPsbA (1.1%). One reason for the differences in Chl *f* production might be the levels of srPSII that accumulate in each strain. By contrast, the P_*psbA2*__ChlF^7203^/ΔPsbA mutant with the least Chl *f* (0.1% Chl *f*/Chl *a*) did not show a distinct emission band above 700 nm. A nearly linear correlation was observed when the relative intensity of the ~720 nm fluorescence peak (expressed as F_~720 nm_/F_~685 nm_) was plotted against the corresponding Chl *f*/Chl *a* level in the different mutants (Figure 2b). These findings underscore the utility of room‐temperature fluorescence spectroscopy as a rapid method for assaying Chl *f* levels in ChlF mutants.

### 3.5 Isolation and characterization of Flag‐tagged ChlF^7521^

There is still uncertainty about whether the active form of ChlF is a homodimer (Shen et al., 2019) or a monomeric PSII‐like srPSII complex (Trinugroho et al., 2020). To investigate the possibility that the observed discrepancy results from the expression of different types of ChlF subunit in the respective studies – ChlF^7521^ in the case of Shen et al. (2019) and ChlF^7203^ in the case of Trinugroho et al. (2020) – we decided to isolate 3xFlag‐tagged ChlF^7521^ complexes from the P_*cpc560*__Flag‐ChlF^7521^/ΔPsbA mutant via anti‐Flag affinity chromatography.

**FIGURE 3:**
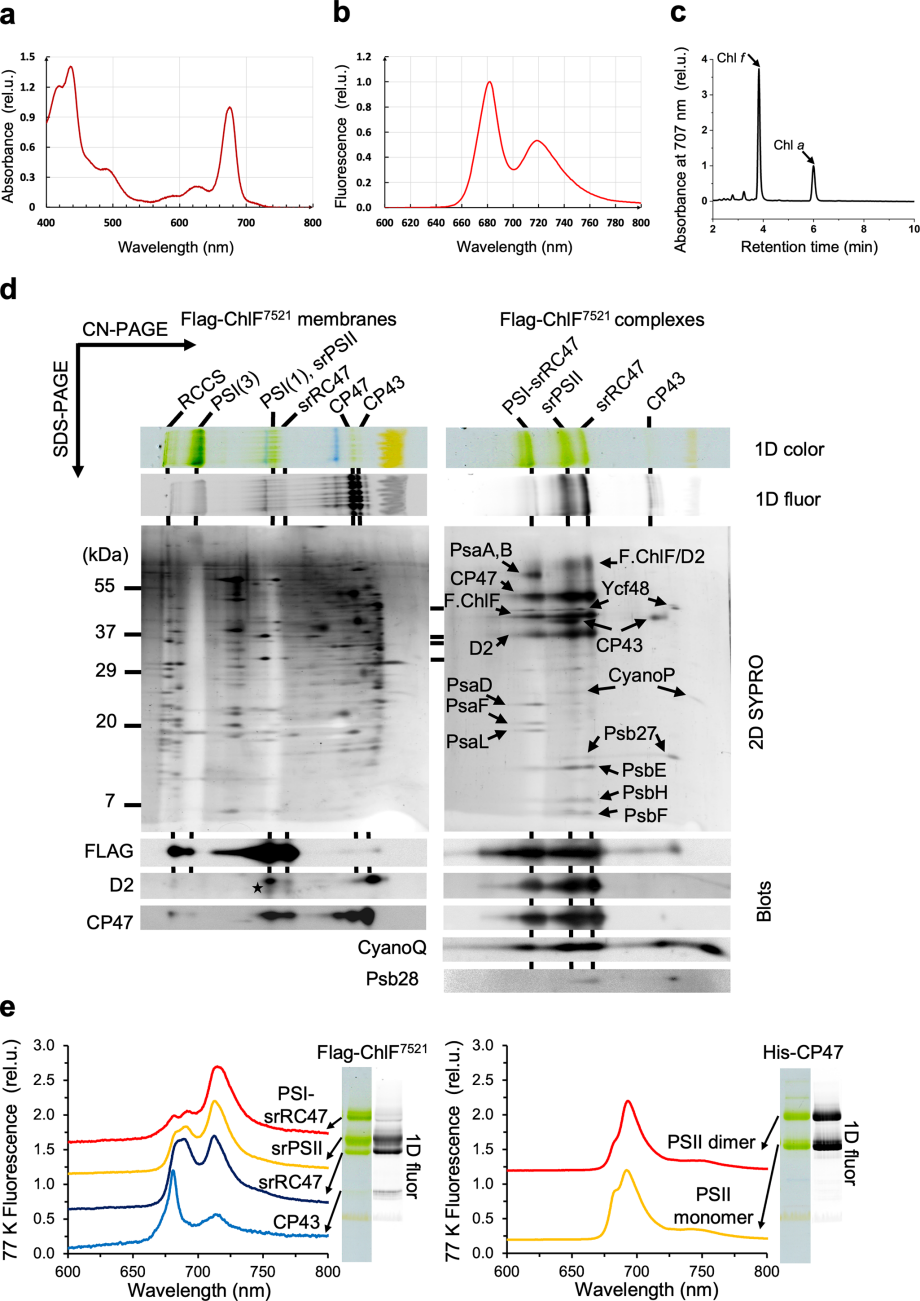
**Characterization of Flag‐ChlF** ^**7521**^ **complexes**. Room‐temperature absorption **(a)** and fluorescence (**b**) spectra of isolated Flag‐ChlF^7521^ complexes. **c**, HPLC elution profile at 707 nm for pigments extracted from the Flag‐ChlF^7521^ complexes. **d**. Two‐dimensional (2D) gel electrophoresis of thylakoid membranes (left) and Flag‐ChlF^7521^ complexes (right) isolated from the P_*cpc560*__Flag‐ChlF^7521^/ΔPsbA mutant. Complexes were initially separated by clear‐native (CN) PAGE in the first dimension (1D color), visualized by fluorescence at room‐temperature (1D fluor) and then denatured to separate individual proteins within complexes by SDS‐PAGE in the second dimension. Proteins were visualized by SYPRO orange staining (2D SYPRO). FLAG‐ChlF and D2 in thylakoid membranes were identified by immunoblots, while proteins in the Flag‐ChlF^7521^ complexes were assigned according to Trinugroho et al. (2020) and immunoblots. Note, the D2 blot was done after the Flag blot on the same membrane, leading to crossover signals (star) from the Flag blot to the D2 blot. Abbreviations: RCCS, super‐complex of PSI and PSII; PSI(3), PSI trimer; PSI(1), PSI monomer; F.ChlF, Flag‐ChlF; F.ChlF/D2, Flag‐ChlF and D2 aggregates; PsaA,B; PsaA or PsaB proteins; U.P., unassembled proteins. **e**, In‐gel 77 K fluorescence of the Flag‐ChlF^7521^ complexes (left) and a PSII sample (right) isolated from a *Syn*6803 His‐CP47 strain. The 1D fluor was shown for comparison. It is noteworthy that there is a systematic 3–5 nm blue‐shift of the Chl *f* peak measured in‐gel at 77 K compared to room‐temperature (see Figure 3b). rel.u., relative unit.

Thylakoid membranes isolated from the mutant were solubilized by DDM, and Flag‐tagged ChlF^7521^ complexes were isolated by affinity chromatography using an anti‐Flag resin (MATERIALS AND METHODS). The various fractions obtained during purification—comprising solubilised thylakoid membranes, unbound fraction, the first and last washes of the resin, and the final eluate—were collected and subjected to room‐temperature absorption and fluorescence spectroscopy (Figure S8). Analysis of the final eluate revealed a Qy peak at 675 nm and an absorption shoulder above 700 nm (Figure 3a). HPLC confirmed the presence of Chl *f* in the final eluate at a level of 2.4% Chl *f*/Chl *a*, which is likely responsible for the distinctive room‐temperature fluorescence peak at 718 nm (Figure 3b,c).

Two‐dimensional (2D) gel electrophoresis combined with immunoblotting was used to analyse the Flag‐tagged ChlF^7521^ complexes formed in *Syn*6803 (Figure 3d). Flag‐tagged ChlF^7521^ was predominantly found in the thylakoid membrane in a complex of similar size to monomeric PSII, and to a lesser extent, in larger super‐complexes. Analysis of the final affinity‐purified preparation confirmed that the Flag‐tagged ChlF^7521^ preparation was highly similar in composition to the Flag‐tagged ChlF^7203^ preparation described by Trinugroho et al. (2020). Three types of complexes were detected. The first was the srPSII complex encompassing Flag‐ChlF^7521^, D2, CP43, CP47, low‐molecular‐mass PSII subunits PsbE, PsbH, and PsbF, but lacking the extrinsic subunits (PsbO, PsbU and PsbV) associated with oxygen‐evolving PSII. Trace amounts of CyanoQ could be detected by immunoblotting (Figure 3d). Additionally, some accessory factors, assigned as Ycf48, CyanoP and Psb27, were detected at sub‐stoichiometric levels. The second was the srRC47 complex representing srPSII lacking CP43 (with some detached during electrophoresis) and the third was a PSI‐srRC47 complex, comparable in size to the PSII dimer. These findings closely align with the observations from Trinugroho et al. (2020), except for the enhanced abundance of the PSI‐srRC47 complex in the preparation. Importantly, our investigation did not reveal any evidence supporting the presence of a ChlF homodimer.

In‐gel 77 K fluorescence analysis of the various complexes (Figure 3e) revealed a distinct peak at 712–713 nm in both the srPSII and srRC47 complexes, assigned to Chl *f*, along with two peaks at 685 nm and 691 nm, similar to that observed in a *Syn*6803 PSII sample, attributed to Chl *a*. The Chl *f* peak in srRC47 displayed a lower amplitude than that in srPSII, suggesting the presence of Chl *f* at the interface of CP43 and ChlF or within CP43. The free CP43 module also exhibited a fluorescence peak above 700 nm, indicating the presence of Chl *f* in CP43, albeit at relatively low levels compared with other complexes. The broader and higher fluorescence peak at 715 nm observed in the PSI‐srRC47 complex is likely due to contributions from both Chl *f* and Chl *a* present in PSI. Overall, these spectra are consistent with those observed by Trinugroho et al. (2020) but with a notably higher amplitude of the Chl *f* peak in all components, which can be attributed to the higher Chl *f* content of the preparation.

## 4 DISCUSSION

In this study, we have substantially enhanced Chl *f* production in *Syn*6803, achieving a Chl *f* to Chl *a* ratio of 5–8% in both the ΔPsbA and WT backgrounds‐ a level comparable to native species (Gan et al., 2014a). This was accomplished by employing a strong P_*cpc560*_ promoter, an N‐terminally truncated variant of ChlF^7203^ and growing cultures at low‐light intensities. Further enhancement may be achieved by optimizing the light quality as done for *Syn*7002 (Shen et al., 2019). To enhance FRL absorbance, particularly in WL photosystems that may lack a preference for binding Chl *f* over Chl *a*, the increased production of Chl *f* could prove crucial for its incorporation into the photosynthetic apparatus. Tros and colleagues have reported the successful incorporation of Chl *f* into WL photosystem I in *Syn*7002. Their study demonstrated a direct correlation between the levels of Chl *f* in cells and the numbers of Chl *f* bound to WL PSI. Moreover, as the Chl *f* content increased, there was a corresponding enhancement in FRL absorbance (Tros et al., 2021).

We have also provided evidence that ChlF^7251^ expressed in *Syn*6803 assembles into a srPSII complex similar in subunit composition to the srPSII complex isolated by Trinugroho et al. (2020) following expression of ChlF^7203^. One difference is the increased level of a PSI/RC47 complex in the final preparation, which may be an artefact of the isolation procedure as discussed recently for a PSI/RCII complex purified by Zhao and colleagues (Zhao et al., 2023) or potentially, a complex involved in the transfer of Chl *f* to PSI. In contrast, Shen et al. (2019) have reported that ChlF^7251^ expressed in *Syn*7002 assembles into a homodimeric complex, not a larger srPSII complex, and that Chl *f* can still be produced in a D2 knock‐out mutant, at odds with the results obtained with *Syn*6803 (Trinugroho et al., 2020). The reasons for these conflicting results remain unclear.

Several challenges remain for achieving FRL‐driven photosynthesis in heterologous systems. Cyanobacteria undergoing far‐red light photoacclimation (FaRLiP) synthesize not only Chl *f* and Chl *d* but also a new photosynthetic apparatus (FRL‐PSI, FRL‐PSII, FR‐APC)), encoded by a ~ 21 gene FaRLiP cluster in *C. thermalis* 7203, to incorporate these novel FR pigments for FRL photosynthesis (Gan et al., 2014b). While WL photosystem I (PSI) can bind heterologously produced Chl *f*, there is significantly less red‐shifted Chl *f* absorption in WL‐PSI, probably due to the lack of FRL specific residues found in FRL‐PSII and FRL‐PSI needed to fine‐tune the optical properties of the FR pigment (Tros et al., 2021). Besides, although introducing Chl *f* in *Syn*6803 might enhance FRL capture, the absence of Chl *d* in the PSII reaction centre may impede FRL‐driven charge separation (Gisriel et al., 2023, 2022). Moreover, the introduction of FRL‐APCs to form an additional FRL antenna may be important to increase the FRL absorption and quantum efficiency of FRL photosynthesis (Mascoli et al., 2022).

To address these challenges, future experiments will focus on expressing the entire FR‐PSI, FR‐PSII and FR‐APC cluster in heterologous systems; engineering the endogenous WL‐PSI and WL‐PSII complexes of non‐FaRLiP strains to bind Chl *f* and elucidating the nature of Chl *d* synthesis in FaRLiP species to allow heterologous Chl *d* expression.

## AUTHOR CONTRIBUTIONS

P. J. N. conceived the research; M. Q., H. N. T., M. B., Z. X., J. T. performed the experiments; all authors analysed the data and M. Q., H. N. T., P. J. N. contributed to writing of the manuscript. These authors contributed equally: Man Qi and Henry N. Taunt.

## Supporting information


**Supporting information**.

## Data Availability

The data that supports the findings of this study are available in the main text and supplementary material of this article.
