# Supplementary material for: Enhancing the production of chlorophyll f in the cyanobacterium Synechocystis sp. PCC 6803
Source: Physiol Plant. 2025 Mar 26;177(2):e70169. doi: 10.1111/ppl.70169 (PMC11946780; doi:10.1111/ppl.70169)
Supplement: Supplementary file 1 — Supporting information. [file PPL-177-e70169-s001.pdf]

Supplementary Figures for

Enhancing the production of chlorophyll *f* in the cyanobacterium *Synechocystis* sp. PCC 6803

Man Qi, Henry N. Taunt, Martina Bečková, Zhi Xia, Joko P. Trinugroho, Josef Komenda, Peter J. Nixon

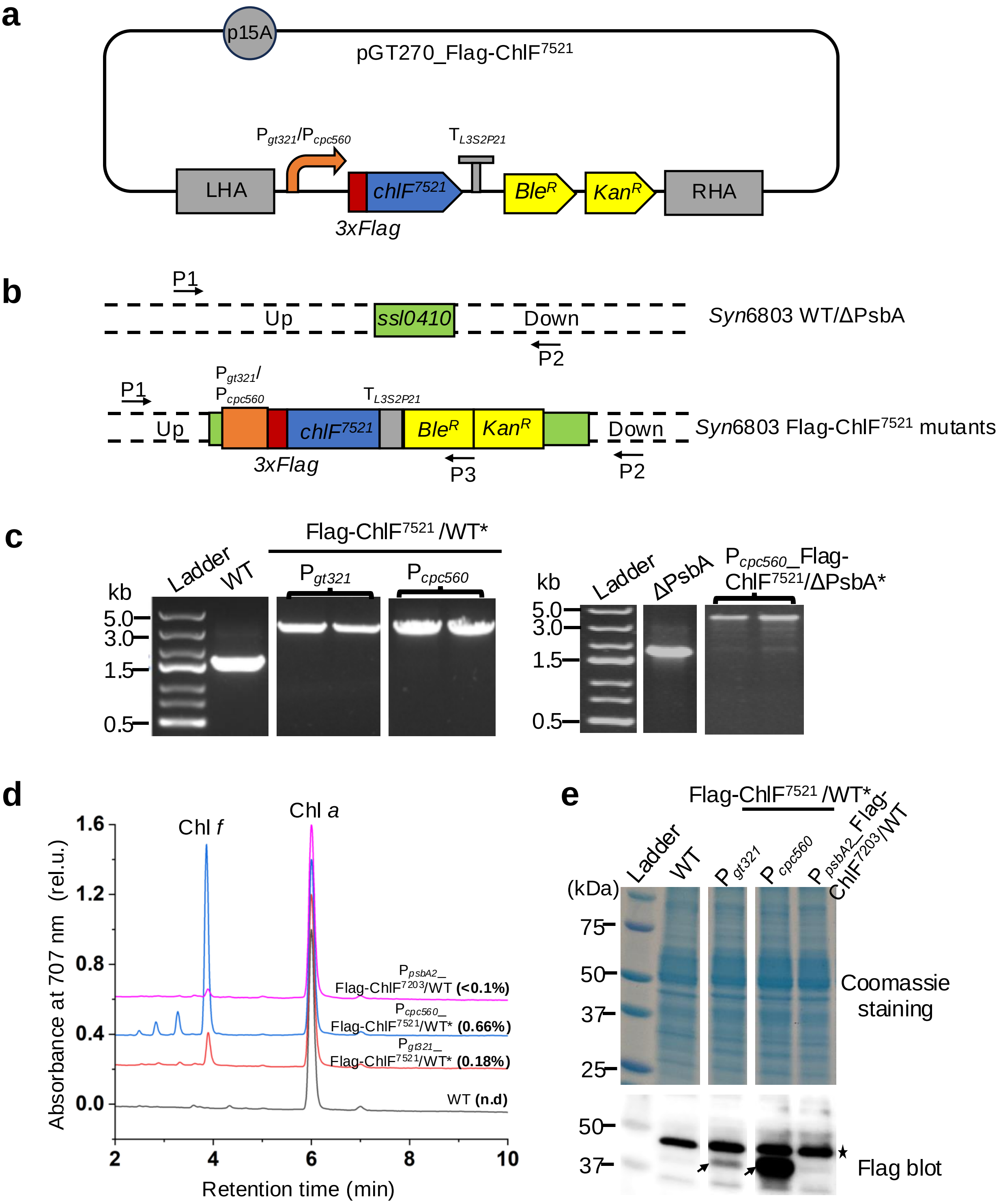

Fig. S1 Testing different promoters for Chl *f* production in *Syn*6803. a, Cartoon illustrating the composition of the pGT270_Flag-ChlF^{7521} vector with either the P$_{gt321}$ promoter or the P$_{cpc560}$ promoter. LHA, left homologous arm; RHA, right homologous arm. **b**, The *ssl0410* locus of the *Syn*6803 WT/ΔPsbA strain and the resulting Flag-ChlF^{7521} mutants following transformation of the pGT270_Flag-chlF^{7521} vectors. Binding sites of the primers (Table S2) used for PCR genotyping were indicated. **c**, Agarose gel of the PCR fragments confirming the genotypes of the Flag-ChlF^{7521} mutants. Parental WT and ΔPsbA strains are predicted to give an PCR fragment of 1.7 kb while for P$_{gt321}$_Flag-ChlF^{7521} /WT* mutant, it is 3.2 kb, and for P$_{gt321}$_Flag-ChlF^{7521} /WT* and P$_{gt321}$_Flag-ChlF^{7521} /ΔPsbA*, it is 3.7 kb. **d**, HPLC elution profiles at 707 nm for pigments extracted from various ChlF mutants grown in BG11 with 5 mM glucose (calculated Chl *f*/Chl *a* levels were indicated in bold parentheses). A WT transformant expressing the Flag-ChlF^{7203} at the *psbA2* locus of *Syn*6803 genome (P$_{psbA2}$ _Flag-ChlF^{7203}/WT mutant), obtained from Trinugroho et al. (2020), was used for comparison here. rel.u., relative unit; n.d, not detected. **e**, SDS-PAGE and immunodetection of Flag-ChlF in thylakoid membranes extracted from various ChlF mutants. 3 µg of Chl was loaded per lane. The black arrows indicated the position of FLAG-ChlF protein while the star represents an unrelated cross-reaction. Note, these lanes were from the same SDS-PAGE gel but not placed next to each other.

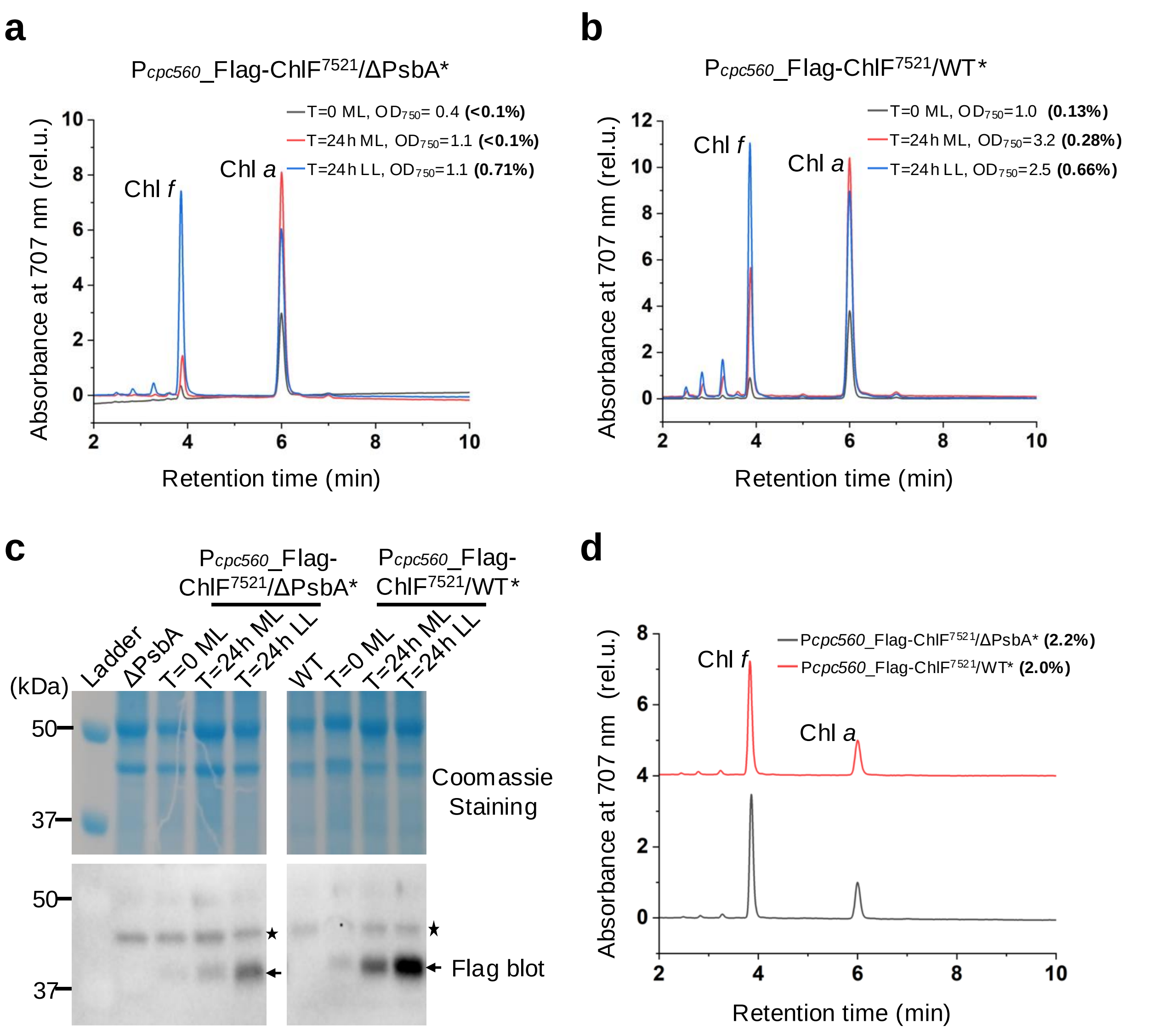

Fig. S2 Low light confers an advantage for Chl *f* and ChlF accumulation in Syn6803. a,**b**, HPLC elution profiles at 707 nm for pigments extracted from P_{cpc560}_Flag-ChlF^{7521}/ΔPsbA* cells (**a**) and P_{cpc560}_Flag-ChlF^{7521}/WT* cells (**b**) grown at different light intensities (calculated Chl *f*/Chl *a* levels in cells are indicated in bold parentheses). Pigments were extracted from the same volume of cells. Cultures were grown in BG11 with 5 mM glucose under medium light (ML, 20-35 μmol photons $m^{-2}s^{-1}$) to OD_{750} 0.4-1.0, resulting in a basal level of Chl *f* (T=0 ML). Then the cultures were split into half, with one incubated under the same ML condition for 24 hours (T=24h ML) and the other moved to low light (LL, 2-10 μmol photons $m^{-2}s^{-1}$) for 24 hours (T=24h LL). Cells grown under ML and LL for 24 hours both synthesize new Chl *a* and Chl *f*, but Chl *f* synthesis under LL is more pronounced compared to that under ML, despite more Chl *a* synthesis under ML. **c**, SDS-PAGE and immunodetection of ChlF. The black arrows indicate the position of FLAG-ChlF protein while the star represent unspecific cross-reactions. d, HPLC elution profiles at 707 nm for pigments extracted from P_{cpc560}_Flag-ChlF^{7521}/ΔPsbA* and P_{cpc560}_Flag-ChlF^{7521}/WT* cells grown under continuous LL for 4 days (OD_{750}=2.6-2.7).

```
ChlF_7335     ---------------------MIQTGFGRT----SALEGF----EQPFDPAQAIDLESPL 31
D1_Syn6803    ------------------------------------------------------------ 0
ChlF_7203     ---------------------------★MVSKTDSAIATPT------------------- 13
ChlF_7507     MQKIILLSAVLETVAIENSYLIMQGSINMTPKSDSIIATSTVSVDKAVKAAAPANVNCHG 60
ChlF_7521     ----------------------------MKLESDHVIATSD------------------- 13
ChlF_9212     ----------------------------MKLESDHVIATSD------------------- 13

ChlF_7335     TSTDTSVENTTRNAGALWPSSQPLSPWERFCRWVTSTENRIYIGWFGMLAIPTLATAAIV 91
D1_Syn6803    ------------★MTTTLQQRESASLWEQFCQWVTSTNNRIYVGWFGTLMIPTLLTATTC 47
ChlF_7203     ----RGDPTEQIPVTNELKKRQSTSIWDRFCNWVTSTENRLYIGWFGVLMIPCMLTAASV 69
ChlF_7507     AGVAPANQNLQTIATNILERWEEVSLWEKFCSWVTSIENRLYVGWFGILMIPTILTATTV 120
ChlF_7521     ----SSNYTSEPTANKLSERRKKVNHWEKFCSWVTSTENRLYVGWFGVLMIPCILTATTV 69
ChlF_9212     ----SSDYTSEPTANKLSKRRKKVNYWEKFCSWVTSTENRLYVGWFGVLMIPCVLTAATV 69
                                        :   . *::** **** :**:*:**** * ** : **:

ChlF_7335     FVLAIIAAPAVDMDGTGRMVSGSLLDGNNLITAAVVPTSAAIGLHFYPIWEAASLDEWLI 151
D1_Syn6803    FIIAFIAAPPVDIDGIREPVAGSLLYGNNIISGAVVPSSNAIGLHFYPIWEAASLDEWLY 107
ChlF_7203     FIVAIIAAPAVDMDGMSSPITGSLLDGNNIITAAVVPTSAAIGLHFYPLWEAASLDEWLY 129
ChlF_7507     FIIAFIAAPPVDMDGMGSPISGALLDGNNIISAAVVPTSDAIGLHFYPIWEAASLDEWLY 180
ChlF_7521     FIIAIIAAPPVDMDGIGAPISGSILSGNNIITAAVVPTSAAIGLHFYPIWEAVSIDEWLY 129
ChlF_9212     FIIAIIAAPPVDMDGIGVPISGSILSGNNIITAAVVPTSAAIGLHFYPIWEAASIDEWLY 129
              *::*:**** **:**    ::*::* ***:*:.****:* ********:***.*:****

ChlF_7335     NGGPYQLIVLHFIIGIISYQDREWELSYRLKMRPWISLAFTAPVAASVSVLLVYPVGQGG 211
D1_Syn6803    NGGPYQLVVFHFLIGIFCYMGRQWELSYRLGMRPWICVAYSAPVSAATAVFLIYPIGQGS 167
ChlF_7203     NGGPYQLIVLHFLIGIICYQDREWELSYRLGMRPWISLAFTAPVAASISVFLVYPVGQGS 189
ChlF_7507     NGGPYQMIVLHFLISIICYQDREWELSYRLGMRPWISLAFTAPVAAAISVFLIYPIGQGS 240
ChlF_7521     NGGPYQMIVLHFLIGIIAYQDREWELSYRLGMRPWISLAFTAPVAAAVSVLLIYPVGQGS 189
ChlF_9212     NGGPYQLIVLHFLIGIIAYQDREWELSYRLGMRPWISLAFTAPVAASVSVLLIYPVGQGS 189
              ******::*:**:*.*:.* .*:******* *****.:*::***:*: :*:*:**:***.

ChlF_7335     FASGMPLGISGTFTFMMQFQADHNILASPLHQMGVIGVLGGALLCAVHGSLVTSTVCRAP 271
D1_Syn6803    FSDGMPLGISGTFNFMIVFQAEHNILMHPFHMLGVAGVFGGSLFSAMHGSLVTSSLVRET 227
ChlF_7203     FSAGMPLGISGTFNFMLRFQADHNILMSPFHVLGVIGVLGGAFLCAMHGSLVTSTLIRMD 249
ChlF_7507     FSAGMPLGIAGTFNFMFQFQADHNILMSPLHQLGVIGVLGGAMMSAMHGSLVTSTLIRTK 300
ChlF_7521     LSAGMPLGISGTFHFMLQFQADHNILMSPLHQLGVIGVLGGAFAAAMHGSLVTSTLIRSH 249
ChlF_9212     LSAGMPLGISGTFHFMLQFQADHNILMSPLHQLGVIGVLGGAFAAAMHGSLVTSTLIRSH 249
              :: ******:*** **: ***:****  *:* :** **:**:: .*:*******:: *

ChlF_7335     AQTMALTTTKTGTDRQKPKKAKTYSFEHAQAYQQTLLWRGAKFNSSRAVHFCLAALPVAG 331
D1_Syn6803    -----TEVESQNYGYKFGQEEETYNIVAAHGYFGRLIFQYASFNNSRSLHFFLGAWPVIG 282
ChlF_7203     GDRSDELSESTNAGYKLGQKRPTYSFRAAQLYLWRLIWRGTSFPNSRRLHFFLAAFPVAG 309
ChlF_7507     -E---SNSESINAGYKLGQKHPTYNFKSAQFYLGRLGWRRASFPNSRKLHFFLAAFPVAG 356
ChlF_7521     NH---SESESINKGYKLGQQHPTYNFRSAQVYLWHLIWHRVSFPNSRKLHFFLAALPVAG 306
ChlF_9212     NH---SESESINKGYKLGQQHPTYNFRSAQVYLWHLIWQRVSFPNSRKLHFFLAALPVAG 306
                        . . . :  ::  **.:  *: *   * :: ..* .** :** *.* ** *

ChlF_7335     IWSAAIGVDLAAFDFDRLSFELPSHISVRKTVVPTWSDVVNQANLGIHTVGEKTPPKFSE 391
D1_Syn6803    IWFTAMGVSTMAFNLNGFNFNQS-ILDSQGRVIGTWADVLNRANIGFEVMH--ERNAH-- 337
ChlF_7203     IWSAALGVDIAAFNFEKLNFEPT-HIESQGRTVNTWADAIDWANLGIDMAR--DRQLH-- 364
ChlF_7507     IWSAALGVDIAAFNLEKLTFEQP-EITSQGRVIHTWSDTIDWANLGIKVVGESDRQVY-- 413
ChlF_7521     IWSAALGVDIAAFDFDYLQFHQP-EIKSQGQIIHTWADTIDWASLGIKILD--ERHIY-- 361
ChlF_9212     IWSAALGVDIAAFDFDYLQFHQP-ELKSQGQIIHTWADTIDWASLGIKVLD--ERHIY-- 361
              ** :*:**.  **::: : *.    :  :   : **:*.:: *.:*:.         .

ChlF_7335     SGFPEFKLSEFVEPIAEDSASTLLSPHS--     419
D1_Syn6803    -NFPLDLASGEQAPVALT------APAVNG     360
ChlF_7203     -QFPSDLMAVSNE-----------------     376
ChlF_7507     -NFSENFTTGEAVPLSFE------F-----     431
ChlF_7521     -DFPENLTAGEVVPWK--------------     376
ChlF_9212     -DFPENLTAGEVVPWK--------------     376
                *     :
```

Fig. S3 Multiple sequence alignment of various ChlF proteins with the conventional D1 protein in *Syn*6803. The N-terminal region before the first transmembrane helix of D1 is shown in black frame. The red stars highlight the start of D1_Syn6803 and ChlF_7203 sequences. Sequences used were taken from Cardona et al. (2015). D1_Syn6803, conventional D1 from *Synechocystis* sp. PCC 6803 (WP_010871214.1); ChlF_7335, ChlF from *Synechococcus* sp. PCC 7335 (WP_006456314.1); ChlF_7203, ChlF from *Chroococcidiopsis thermalis* PCC 7203 (WP_015153111.1); ChlF_7507, ChlF from *Calothrix* sp. PCC 7507 (WP_015126592.1); ChlF_9212, ChlF from *Chlorogloeopsis fritschii* PCC 9212 (WP_016873418.1).

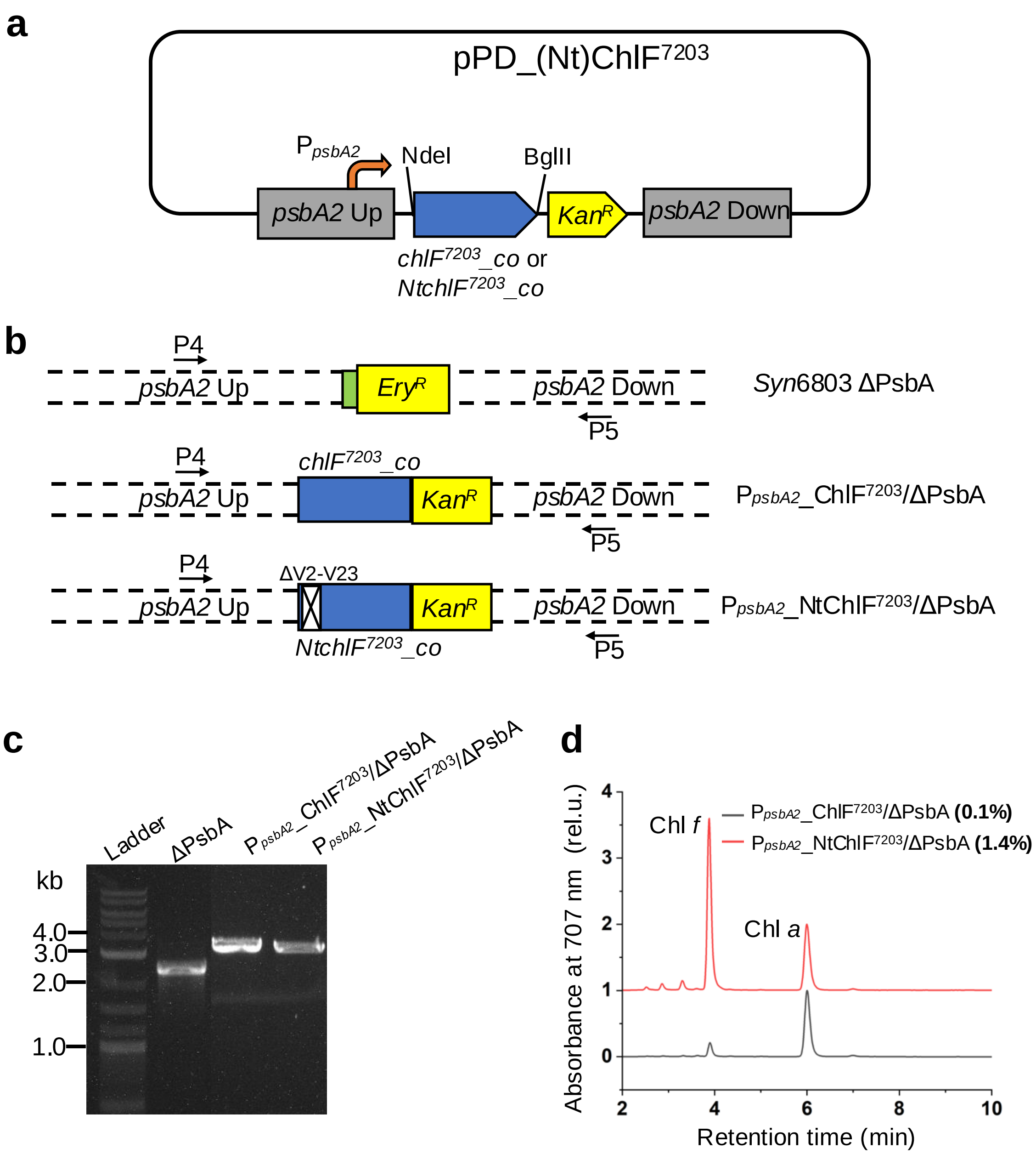

Fig. S4 A N-terminal truncated ChlF[7203] produces more Chl *f* compared to ChlF[7203]. a, Cartoon showing the composition of the pPD_(Nt)ChlF[7203] vector used to express either full length ChlF[7203] or a N-terminal truncated ChlF[7203] lacking residues V2 to V23 (NtChlF[7203]). NdeI and BglII restriction sites allowing the insertion of *chlF* genes are shown. *chlF[7203]_co* and *NtChlF[7203]_co*: codon-optimized *chlF[7203]* and *NtchlF[7203]* genes; *psbA2* Up and down, nucleotide sequences located upstream (Up) /downstream (Down) the *psbA2* coding sequence (CDS) in *Syn*603; *Ery[R]*, erythromycin-resistance gene. **b**, The *psbA2* locus of the *Syn*6803 ΔPsbA strain and the resulting ChlF[7203] mutants following transformation of the pPD_(Nt)ChlF[7203] vector. Binding sites of the primers (Table S2) used for PCR genotyping are indicated. **c**, Agarose gel of the PCR fragments confirming the genotypes of the ChlF mutants. Parental ΔPsbA strain is predicted to give an PCR fragment of 2.4 kb while for P_{psbA2}_ChlF[7203]/ΔPsbA mutant and P_{psbA2}_NtChlF[7203]/ΔPsbA mutant, it is 3.2 kb. **d**, HPLC elution profiles at 707 nm for pigments extracted from the ChlF mutants grown in BG11 with 5 mM glucose under continuous low light (2-10 µmol photons m^{-2} s^{-1}) to stationary phase (OD_{750} at approximately 2). The calculated Chl *f*/Chl *a* levels in mutants are indicated in bold parentheses. rel.u., relative unit.

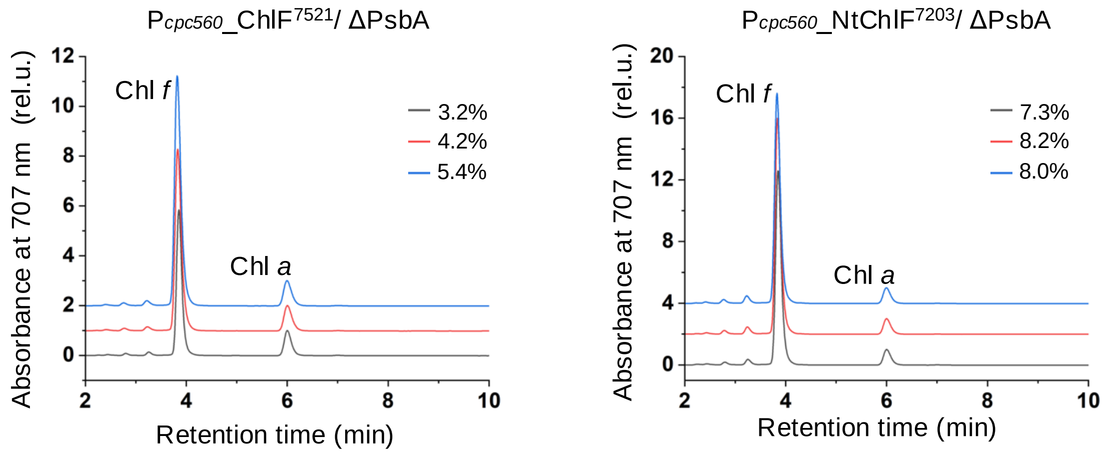

Fig. S5 HPLC elution profiles at 707 nm for pigments extracted from the P*cpc560*_ChlF[7521]/ΔPsbA and P*cpc560*_NtChlF[7203]/ ΔPsbA mutants. Each mutant is grown in BG11 with 5 mM glucose, in triplicate, to stationary phase (OD_{750} at approximately 2) under low light (<5 μmol photons m^{-2} s^{-1}). The HPLC elution profiles include three traces for each mutant, corresponding to biological replicates. Each trace is labeled with the calculated Chl *f*/Chl *a* level.

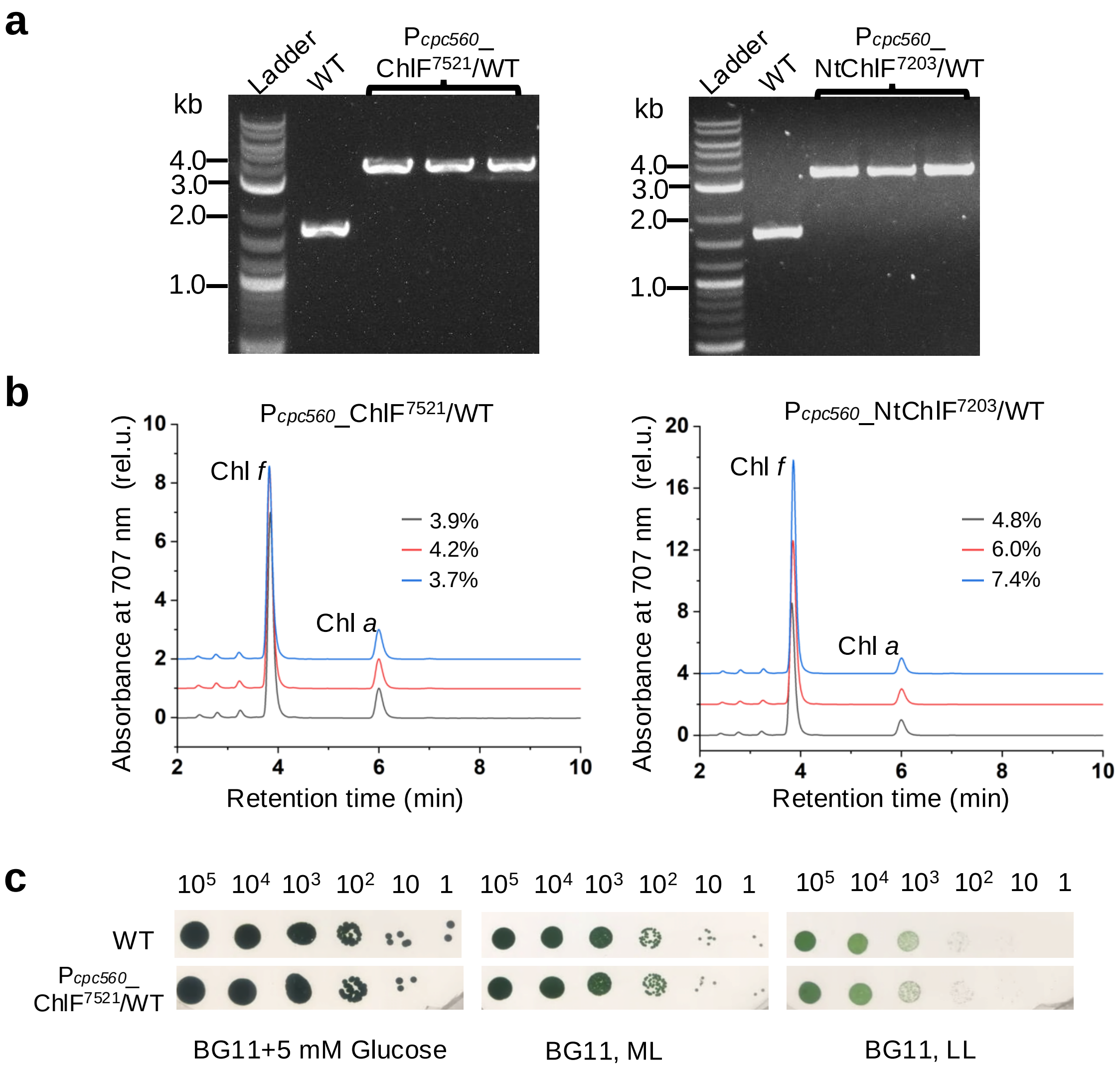

Fig. S6 Generation and characterization of ChlF mutants in the *Syn*6803 WT background. a, Agarose gel of the PCR fragments confirming the genotypes of the P_{cpc560}_ChlF^{7521}/WT and P_{cpc560}_NtChlF^{7203}/WT mutants. Primers used for genotyping were the same as indicated in Fig 1b. Parental WT strain is predicted to give an PCR fragment of 1.7 kb while for ChlF mutants, it is 3.5-3.6 kb. **b**, HPLC elution profiles at 707 nm for pigments extracted from the P_{cpc560}_ChlF^{7521}/WT and P_{cpc560}_NtChlF^{7203}/WT mutants. Each mutant is grown in BG11 with 5 mM glucose, in triplicate, to stationary phase (OD_{750} at approximately 2) under low light (2-10 μmol photons m^{-2} s^{-1}). The HPLC elution profiles include three traces for each mutant, corresponding to biological replicates. Each trace is labeled with the calculated Chl *f*/Chl *a* level. The HPLC traces are labelled with their corresponding calculated Chl *f*/Chl *a* levels. **c**, Photoautotrophic growth test of the P_{cpc560}_ChlF^{7521}/WT mutant under medium light irradiation (ML, 20-35 μmol photons m^{-2} s^{-1}) and low light irradiation (LL, 2-10 μmol photons m^{-2} s^{-1}).

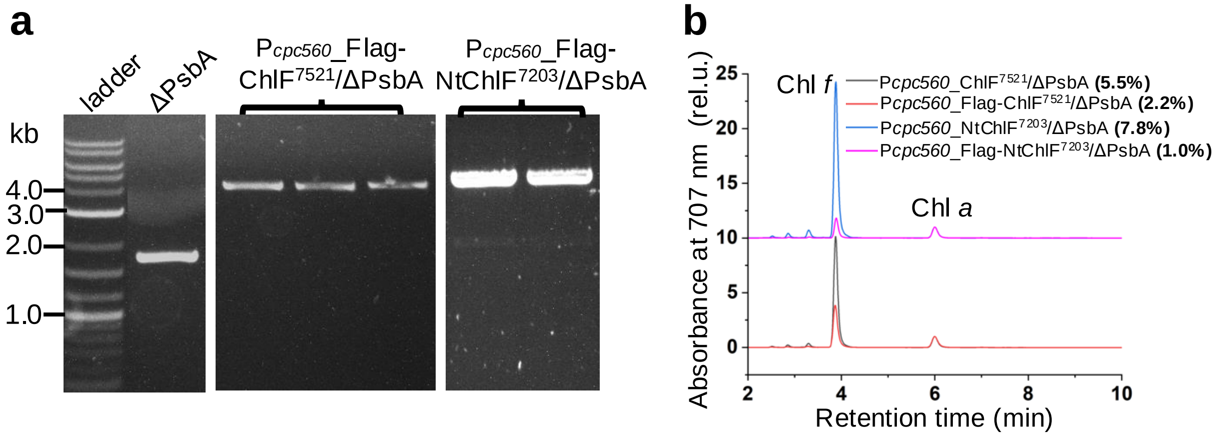

Fig. S7 Addition of a N-terminal 3xFlag tag to ChlF impairs Chl f production. a, Agarose gel of the PCR fragments confirming the genotypes of the ChlF mutants. Primers used for genotyping were the same as indicated in Fig 1b. Parental ΔPsbA strain is predicted to give an PCR fragment of 1.7 kb while for P_{cpc560}_Flag-ChlF^{7521}/ΔPsbA mutant, it is 3.7 kb, and for P_{cpc560}_Flag-NtChlF^{7203}/ΔPsbA mutant, it is 3.6 kb. **b**, HPLC elution profiles at 707 nm for pigments extracted from various ChlF mutants grown in BG11 with 5 mM glucose under continuous low light (2-10 μmol photons m^{-2} s^{-1}) to stationary phase (OD_{750} at approximately 2). The calculated Chl *f*/Chl *a* levels in mutants were indicated in bold parentheses. rel.u., relative unit.

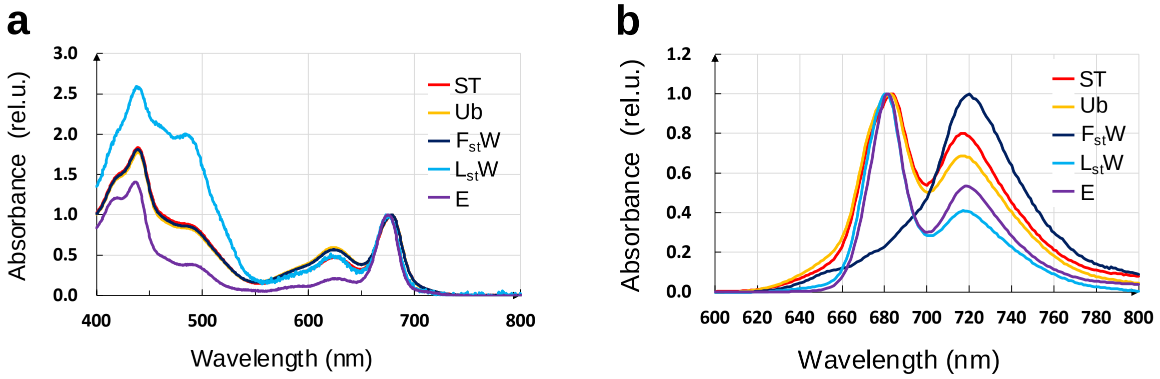

Fig. S8 Room-temperature absorption (a) and fluorescence spectra (b) of different fractions obtained during Flag purification of the P_{cpc560}_Flag-ChlF^{7521}/ΔPsbA mutant. ST, solubilized thylakoid membranes; Ub, unbound fraction; F_{st}W, first wash of the resin; L_{st}W, last wash of the resin; E, the final eluate containing the target proteins. rel.u, relative unit.

Table S1 Sequences of promoters and *chlF* genes used in this study

Parts	Sequences (5'-3')
PGT321	CAGGTGACGTCTATTGACAGCCAGGAGTTACCGAGATATAATGGAATAGGCGCGAGTCAACGTTCCTGAG<br>GAGTGGC
Pcpc560	ACCTGTAGAGAAGAGTCCCTGAATATCAAAATGGTGGGATAAAAAGCTCAAAAAGGAAAGTAGGCTGTGG<br>TTCCCTAGGCAACAGTCTTCCCTACCCCACTGGAAACTAAAAAAACGAGAAAAGTTCGCACCGAACATCAA<br>TTGCATAATTTTAGCCCTAAAACATAAGCTGAACGAAACTGGTTGTCTTCCCTTCCCAATCCAGGACAATCT<br>GAGAATCCCCTGCAACATTACTTAACAAAAAAGCAGGAATAAAATTAACAAGATGTAACAGACATAAGTCC<br>CATCACCGTTGTATAAAGTTAACTGTGGGATTGCAAAAGCATTCAAGCCTAGGCGCTGAGCTGTTTGAGCA<br>TCCCGGTGGCCCTTGTCGCTGCCTCCGTGTTTCTCCCTGGATTTATTTAGGTAATATCTCTCATAAATCCCC<br>GGGTAGTTAACGAAAGTTAATGGAGATCAGTAACAATAACTCTAGGGTCATTACTTTGGACTCCCTCAGTT<br>TATCCGGGGGAATTGTGTTTAAGAAAATCCCAACTCATAAAGTCAAGTAGGAGATTAATTCA
chlF^{7521}	ATGAAGCTAGAGTCAGACCATGTAATTGCAACCTCAGATAGTAGCAATTACACTTCTGAGCCAACAGCAAA<br>CAAACTCTCAGAAAGACGCAAAAAAGTTAATCATTGGGAAAAATTTTGTTCATGGGTTACCAGCACAGAAA<br>ACAGACTATATGTCGGCTGGTTTGGTGTGTTGATGATTCCTTGCATCTTAACAGCAACAACTGTTTTTATCA<br>TCGCCATCATCGCTGCTCCTCCTGTAGACATGGATGGAATAGGTGCGCCCATTTCCGGTTCAATACTTTCT<br>GGAAATAACATTATCACTGCTGCTGTTGTGCCAACATCGGCTGCAATTGGTCTGCATTTTTATCCAATTTGG<br>GAAGCAGTTTCCATTGATGAGTGGCTTTACAATGGTGGGCCATATCAAATGATTGTGCTGCATTTTTTGATT<br>GGCATCATCGCCTATCAGGACCGGGAATGGGAACTAAGTTACCGCTTGGGAATGCGTCCCTGGATTTCTC<br>TAGCATTTACTGCTCCCGTCGCCGCAGCTGTCTCAGTGTTGTTAATCTACCCAGTTGGACAGGGTAGCTTA<br>TCTGCGGGAATGCCTTTAGGAATATCTGGCACATTTCACTTCATGTTGCAGTTTCAAGCAGACCACAACAT<br>CTTGATGAGTCCTTTGCATCAGTTAGGAGTGATTGGGGTTTTAGGTGGTGCTTTTGCGGCTGCAATGCAC<br>GGTTCCTTAGTCACGTCTACCTTAATTCGCAGTCATAATCACAGCGAGTCTGAATCAATTAACAAAGGGTA<br>CAAACTTGGTCAACAACACCCAACCTATAATTTTAGGTCTGCTCAAGTTTATTTATGGCACTTGATATGGCA<br>TCGTGTCAGTTTTCCTAACTCTCGCAAATTGCACTTCTTCTTGGCAGCCTTACCAGTAGCAGGAATTTGGTC<br>TGCGGCTTTGGGTGTGGACATCGCTGCCTTTGACTTTGACTACTTGCAATTTCATCAGCCTGAGATCAAAA<br>GCCAAGGGCAGATTATTCACACTTGGGCAGACACAATTGATTGGGCTTCTTTAGGGATAAAAATTTTAGAT<br>GAACGCCATATTTATGACTTCCCTGAAAACTTAACAGCAGGTGAAGTAGTGCCTTGGAAGTAA
chlF^{7203}*_co*	ATGGTTTCAAAAACAGATTCAGCTATTGCTACACCAACACGTGGTGATCCAACAGAACAAATTCCAGTTAC<br>AAATGAATTAAAAAAACGTCAAAGCACATCAATTTGGGATCGTTTTTGTAATTGGGTTACATCAACAGAAAA<br>TCGTTTATACATTGGTTGGTTTGGTGTTTTAATGATTCCATGTATGTTAACAGCTGCTTCAGTTTTTATTGTT<br>GCTATTATTGCTGCTCCAGCTGTTGATATGGATGGTATGTCATCACCAATTACAGGTTCATTATTAGATGGT<br>AATAACATTATTACAGCTGCTGTTGTTCCAACATCAGCTGCTATTGGTTTACATTTTTATCCATTATGGGAAG<br>CTGCTTCATTAGATGAATGGTTATACAATGGTGGTCCATATCAATTAATTGTTTTACATTTCCTTATCGGCAT<br>TATTTGCTATCAAGATCGTGAATGGGAATTATCATATCGTTTAGGTATGCGTCCATGGATTTCATTAGCTTT<br>TACAGCTCCAGTTGCTGCTTCAATTTCAGTTTTTTTAGTTTATCCAGTTGGTCAAGGTTCATTTTCAGCTGGT<br>ATGCCATTAGGTATTTCAGGTACATTCAATTTTATGTTACGTTTTCAAGCTGATCATAATATTCTGATGTCAC<br>CATTTCATGTTTTAGGTGTTATTGGTGTTTTAGGTGGTGCTTTTTTATGTGCTATGCATGGTTCATTAGTTAC<br>ATCAACTTTAATTCGTATGGATGGTGATCGTTCAGATGAATTATCAGAATCAACAAATGCTGGTTATAAACT<br>TGGTCAAAAACGTCCAACATATTCTTTTCGTGCTGCTCAATTATATCTTTGGCGTTTAATTTGGCGTGGTAC<br>ATCATTTCCAAATTCACGTCGTCTTCATTTTTTTCTGGCTGCTTTTCCAGTTGCTGGTATTTGGTCAGCTGCT<br>TTAGGTGTTGATATTGCTGCTTTTAATTTTGAAAAATTAAACTTTGAGCCAACACACATTGAATCACAAGGTC<br>GTACAGTTAATACATGGGCTGATGCTATTGATTGGGCTAATCTTGGTATTGATATGGCTCGTGATCGTCAA<br>TTACATCAATTTCCATCAGATCTTATGGCTGTTTCAAATGAATAA
NtchlF^{7203}	ATGACTAATGAATTAAAAAAACGCCAAAGCACTAGTATTTGGGATCGCTTTTGTAATTGGGTGACTAGTACT<br>GAAAATCGGCTATATATCGGCTGGTTTGGAGTACTAATGATTCCCTGTATGCTGACAGCAGCCAGCGTGTT<br>TATCGTTGCCATTATTGCCGCACCAGCAGTAGATATGGACGGTATGAGTTCGCCGATTACTGGCTCTCTAC<br>TAGACGGCAATAACATTATTACGGCTGCTGTCGTACCAACATCAGCGGCGATCGGACTGCATTTTTATCCA<br>CTTTGGGAAGCAGCCTCTTTAGATGAGTGGCTCTACAACGGTGGACCTTATCAACTGATCGTGCTGCATTT<br>CTTGATCGGCATTATTTGCTATCAAGATCGCGAATGGGAATTGAGCTATCGCTTGGGAATGCGACCTTGGA<br>TTTCTTTAGCATTTACTGCACCCGTTGCGGCTTCAATTTCGGTCTTTTTAGTCTATCCAGTCGGACAAGGTA<br>GCTTCTCAGCAGGAATGCCACTGGGAATTTCTGGAACTTTCAACTTCATGTTGCGGTTTCAAGCAGACCAT<br>AACATTCTCATGAGTCCTTTTCACGTCTTGGGAGTCATCGGAGTTTTGGGTGGTGCTTTCTTATGCGCTAT<br>GCACGGTTCCCTGGTGACTTCTACCTTGATCCGTATGGATGGCGATCGCAGCGACGAATTGAGCGAATCG<br>ACCAATGCCGGATACAAGCTCGGTCAAAAGCGCCCGACCTACAGTTTTAGGGCAGCTCAACTGTACTTGT<br>GGCGGTTAATTTGGCGGGGAACTAGCTTTCCCAACTCGCGCCGATTGCATTTTTTCCTAGCAGCTTTTCCA<br>GTAGCAGGAATCTGGTCTGCGGCTTTGGGGGTTGACATTGCAGCATTCAACTTTGAAAAGTTGAACTTCGA<br>GCCAACCCACATTGAGAGCCAAGGCAGAACAGTAAATACATGGGCAGACGCGATCGACTGGGCAAATCT<br>AGGCATAGATATGGCACGCGATCGCCAGTTACACCAATTCCCCAGTGACTTGATGGCAGTGAGTAATGAG<br>TAA

Deletion for *NtchlF*^{7203}*_co* gene

Table S2 Sequences of primers used in this study

Primer name	Sequences (5'-3')	notes
	Primers for constructing the vectors	
chlF[7203]_co infu_pPD. F	ACATAAGGAATTATAACCATATGGTTTCAAAAACAGATTCAGC	Forward primer to clone *chlF[7203]_co* into pPD vector
NtchlF[7203]_co infu_pPD F	ACATAAGGAATTATAACCATATGACAAATGAATTAAAAAAACGTCAAAGC	Forward primer to clone *NtchlF[7203]_co* into pPD vector
chlF[7203]_co infu_pPD. R	TGAGTTGAAGGAAGATCTTTATTCATTTGAAACAGCCATAAGATC	Reverse primer to clone *chlF[7203]_co* and *NtchlF[7203]_co* into pPD vector
	Primers for PCR genotyping	
P1	CCGTTCCAATGAAGCG	Primers for genotyping the ChlF mutants transformed with pGT270_Flag-ChlF[7521] vectors or pFly_ChlF vectors
P2	ATGATTGCTTGCAACATTTTG	
P3	ATGGTCTCAAAGATCCATCAATTAATTATTAGTCAAAAAAAAGC	
P4	TTGCGGCTTTAGCGTTCC	Primers for genotyping the ChlF mutants transformed with pPD_(Nt)ChlF[7203] vector
P5	TTCCACCAGATGTCGTTGC	
